# Effects of Overwork

**Published:** 1889-05

**Authors:** 


### Effects of Mental Overwork.

A COMMON FORM OF ILLNESS WHICH IS GENERALLY DISREGARDED.

Some interesting though not novel observations on the symptoms of mental fatigue were discussed at a recent meeting of the Anthropological Society. The result of these investigations goes to prove that weariness of mind, the result of work, like other forms of exhaustion, is recognizable under the two different though related aspects of irritability and incapacity. Further careful inquiry into the same subject would probably show that here, as elsewhere, the former of these conditions is introductory to the latter, and is the natural sequel to that stage of apparently successful overaction which is seen when an organ still fully capable is unduly stimulated.

The observations referred to were culled from a series of reports by school-teachers, and included details of their own sensations as well as of the children under their care. The signs of mental irritability were apparent in sleeplessness and nervous laughter; of fatigue, in sleepiness and incapacity for task work. Lolling, yawning, and a languid manner told that the will was flagging. Headache suggested over strain in study combined with defective ventilation, and perhaps a too sparing diet; while some curious facts bearing on the causation of color-blindness and somnambulism were also noted. Thus, in one case the blue-color perception was for a time obliterated, and the sufferer from this defect found herself painting ivy leaves a bright orange; while in another, a student having retired to rest on the eve of an examination, awoke at his desk to find that he had been busily engaged in drawing humorous cartoons relating to a former conversation. Here we have an instance of cerebral irritation due to overwork which suggests a somewhat close connection between dreaming and somnambulism, and affords a clue to the physiology of the latter condition.

Overwork, both mental and bodily, is at once the most general and the least regarded form of illness to which we are liable in the present age. Do what we may, it is next to impossible to escape from it; but there is, at all events, a certain satisfaction in being able to recognize its features. We must not forget, however, that it is also to a considerable extent a preventable evil, and it is certainly a matter for satisfaction that this fact is not ignored by the reforming party in the legislature. Its treatment in individual cases requires chiefly, that due attention be paid to the two great essentials of timely rest and wholesome diet. Work, however irksome, may, it is generally allowed, be undertaken on a liberal scale if only it be not too continuous, but is broken by timely and adequate intervals of rest. The value of a plain and liberal dietary is hardly less, and we may take it as a maxim for the times that, so long as appetite and sleep are unimpaired there is no dangerous degree of overwork, and, conversely, that a failure in either of these respects should be regarded as a warning signal, to which attention should be paid by relieving the strain of exertion.—*Lancet.*

